# Estimation of heat stress thresholds in lactating Sicilian Cinisara cows under naturally occurring conditions

**DOI:** 10.3168/jdsc.2025-0797

**Published:** 2025-10-03

**Authors:** Carmelo Cavallo, Annalisa Amato, Luigi Liotta, Vincenzo Lopreiato

**Affiliations:** Department of Veterinary Sciences, Messina University, 98168, Messina, Italy

## Abstract

The efficiency of thermoregulation varies among breeds of cattle and can be part of the adaptive ability of local cows, such as Cinisara. This study aimed to assess the resilience to heat stress (HS) of Cinisara cows in a semi-extensive system during summer, evaluating rectal temperature (RT), milk yield (MY) and composition. The study was carried out from June to September 2024 on a commercial dairy farm located in Cinisi (Sicily, Italy) involving 15 primiparous and 20 multiparous lactating Cinisara cows. Temperature-humidity index (THI) was calculated based on temperature and humidity data registered by 3 probes every 15 min. The RT was recorded monthly (once a month) after the morning and evening milking. The MY was recorded monthly and milk samples were collected. Data were analyzed with PROC GLIMMIX and NLIN of SAS. Parity did not affect RT or milk performance response to HS. Overall, the highest RT was registered in August in both morning and afternoon, when THI was above 77 (38.51°C and 39.21°C, respectively). The nonlinear regression (NLIN) analysis revealed a strong and statistically significant relationship between the THI and RT (adjusted R^2^ = 0.89). A THI breakpoint of 77.65 was identified for RT, beyond which RT increased significantly and abruptly by approximately +0.12°C for each unit increase in THI above the threshold. The nonlinear regression analysis applied to MY data revealed a strong relationship between THI and milk production (adjusted R^2^ = 0.90). A THI breakpoint of 76.9 was identified, beyond which MY declined sharply by approximately −8.77 kg/d for each unit increase in THI above the threshold, compared with a much milder decline (−0.69 kg/d for each unit) observed below the breakpoint. This highlights the breed's notable heat tolerance and its suitability for semi-extensive dairy systems in hot climates. Further studies exploring the genetic basis of this adaptation may support conservation and enhancement strategies for native breeds in Mediterranean regions.

## Highlights

• Cinisara cows in pasture cope well with heat stress on milk yield and RT.
• Milk yield and quality remained stable until a THI peak of 77.
• RT and milk yield showed THI thresholds of 77.65 and 76.9, respectively.
• The study supports the genetic potential of Cinisara cows' heat stress resilience.

## Graphical Abstract

**Figure:**
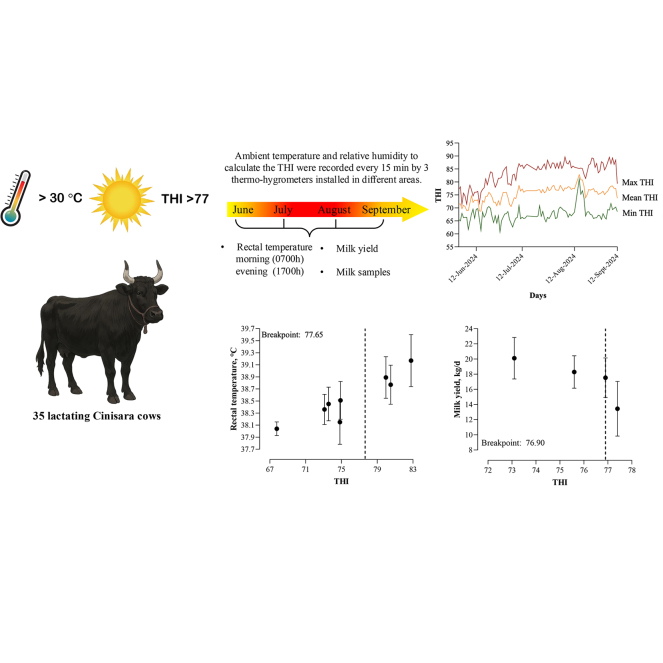
**Summary:** The Cinisara cow is a native Sicilian breed adapted to local environmental conditions. However, few studies have investigated its effective resilience during heat stress conditions. This study aimed to evaluate how increasing temperature-humidity index (THI) during summer affects milk production, milk quality, and body temperature of this breed raised in a semi-extensive system. The results demonstrated that Cinisara cows were able to tolerate higher THI levels compared with commercial breeds such as Holstein cows. Indeed, both rectal temperature (RT) and milk performance were affected when THI exceeded 77. Below this threshold, RT, milk quantity, and quality were only slightly affected by THI. These findings suggest that the Cinisara breed has an effective resilience to high temperatures, likely due to its genetic background and historical management. Thus, supporting and preserving native breeds like the Cinisara could represent a strategy to reduce the impact of climate change on dairy farms.

The pressure of selection for increase milk production is making cows more susceptible to heat stress (**HS**) because the increase of productivity results in increased metabolic heat production (Bernabucci et al., 2010). Moreover, the increased endogenous heat production is caused by the request to consume more feed, and thus by nutrient digestion (Nasr and El-Tarabany, 2017). However, most studies investigating the effects of HS on dairy cows have focused on Holstein dairy cows (Wheelock et al., 2010; Ouellet et al., 2021), aiming to identify out the negative impacts on production, physiology, reproduction, and immunity, as well as evaluating the temperature-humidity index (**THI**) threshold for this breed, which is the most widespread in the world. Nevertheless, previous studies have evaluated differences in THI threshold in Brown Swiss cattle, showing higher thermal tolerance compared with Holstein cows and observing a greater impact on protein yield between the 2 breeds (Maggiolino et al., 2020). These differences are more evident for local breeds, as their production potential is less pronounced due to their genetic and physiological adaptations to living in harsher environments (Rojas-Downing et al., 2017). In the Sicilian context, Cinisara cows are important for the local economy and are adapted to the harsh environment and climate, living in marginal pasture and being efficient in terms of milk quality, even with low-quality feed (Liotta et al., 2011). The Cinisara is a native Sicilian cow that can make productive use of the natural pastures of the hilly and semiarid areas of the Sicilian island. It is a medium-sized cattle breed belonging to the group of Podolica cattle, and it is characterized by a robust skeletal structure and recognized as a distinct local Sicilian ecotype. The breed derives its name from the town of Cinisi, a coastal locality in western Palermo. Morphologically, the Cinisara is distinguished by its black coat, which is often uniform. The Cinisara plays an important role in the conservation of genetic diversity among Sicilian livestock and is valued for its milk, which is used in the making of traditional cheeses. In fact, the Cinisara cow is historically linked to the production of Caciocavallo Palermitano. The cheesemaking production of Caciocavallo Palermitano involves the use of whole and raw cow milk. Considering that Cinisara cow has a low level of management without cooling systems and generally raised under an extensive system, one of the most important factors limiting its production is HS, especially in terms of the resulting economic losses (Tao et al., 2020). No studies have assessed HS response in local cattle breeds such as the Cinisara cow. Summer in Sicily is characterized by a hot-summer Mediterranean climate, with meteorological conditions driven by subtropical high-pressure systems and warm air masses from North Africa. During June, July, and August, average daytime temperatures range between 28°C and 35°C, though heat waves can push maxima above 40°C, particularly in inland valleys. Several authors aimed to point out that local breed cows over time have adapted to specific environments, soil, and climate conditions (Ciotola et al., 2009; Di Gregorio et al., 2017), defining local breeds as “rustic or resistant” compared with worldwide cows such as the Holsteins. But what contributes to the rusticity or resistance of these local cows? We hypothesize that Cinisara cows could have a high critical THI threshold at which they exhibit physiological signs of HS. Thus, our objective was to establish the THI breakpoint for rectal temperature (**RT**) of lactating Cinisara cows and evaluate the response of milk production and its composition over a 90-d period of naturally occurring HS during summer.

The protocol for animal handling and care was reviewed and approved by the University of Messina Animal Care and Use Committee under application number 19/2023. The study was carried out between June and September in a commercial dairy farm located in Cinisi (Sicily, Italy). A total of 35 lactating Cinisara cows, 15 primiparous and 20 multiparous, (DIM: 114 ± 32 d; milk yield: 20.05 ± 2.43 kg/d) were enrolled and monitored over a 90-d period during natural HS exposure in the summer. All cows were raised in a semi-extensive dairy system, grazing most of the time (approximately 18 h/d) and housed in the barn only for milking and feeding (twice a daily). During milking, all cows were individually offered 3 kg of concentrate per head (on DM basis: 17.4% of CP, 5.5% of ether extract, 45.4% of starch, and 6.87% of ash) delivered at 0700 and 1700 h. Before being sent out for grazing, each cow was given ∼3 kg of grass hay (on DM basis: 10.4% of CP, 56.8% of NDF, 33.5% of ADF, and 6.2% of ADL). Ambient temperature and relative humidity to calculate the THI were recorded every 15 min using 3 thermo-hygrometers installed at different locations within the grazing area (Govee IT, model: ‎part_9894). The THI was calculated as follows based on the equation of Thom (1959): THI = (1.8 × T + 32) − [(0.55 − 0.0055 × RH) × (1.8 × T − 26)], where T = ambient temperature (°C) and RH = relative humidity (%). This equation was chosen because it is one of the most widely used in animal science literature, allowing for easier comparison with previous studies. The daily mean, minimum, and maximum THI values recorded during the study period are shown in the Figure 1A. The average THI values at each time point were calculated through the arithmetic mean of the mean THI of 4 consecutive days (the day of measurement and sample collection and the 3 d before). Rectal temperature (°C) was recorded monthly (once a month) after the morning (0700 h) and evening milking (1700 h). The RT was measured using a clinical digital thermometer (TFA Dostmann Thermometer VET 112, Germany) inserted into the rectum. Milk yield (**MY**) was recorded, and milk samples were collected in June, July, and September within the test-day measures. However, milk samples were not collected in August due to a sudden machine breakdown. Milk samples were analyzed for fat, protein, lactose, casein, urea, total milk solids, nonfat milk solids, citric acid, fatty acids, and BHB by mid-infrared spectroscopy (Milkoscan FT2, Foss Electric, Hillerød, Denmark), as well as SCC (Fossomatic, Foss Electric).

**Figure 1:**
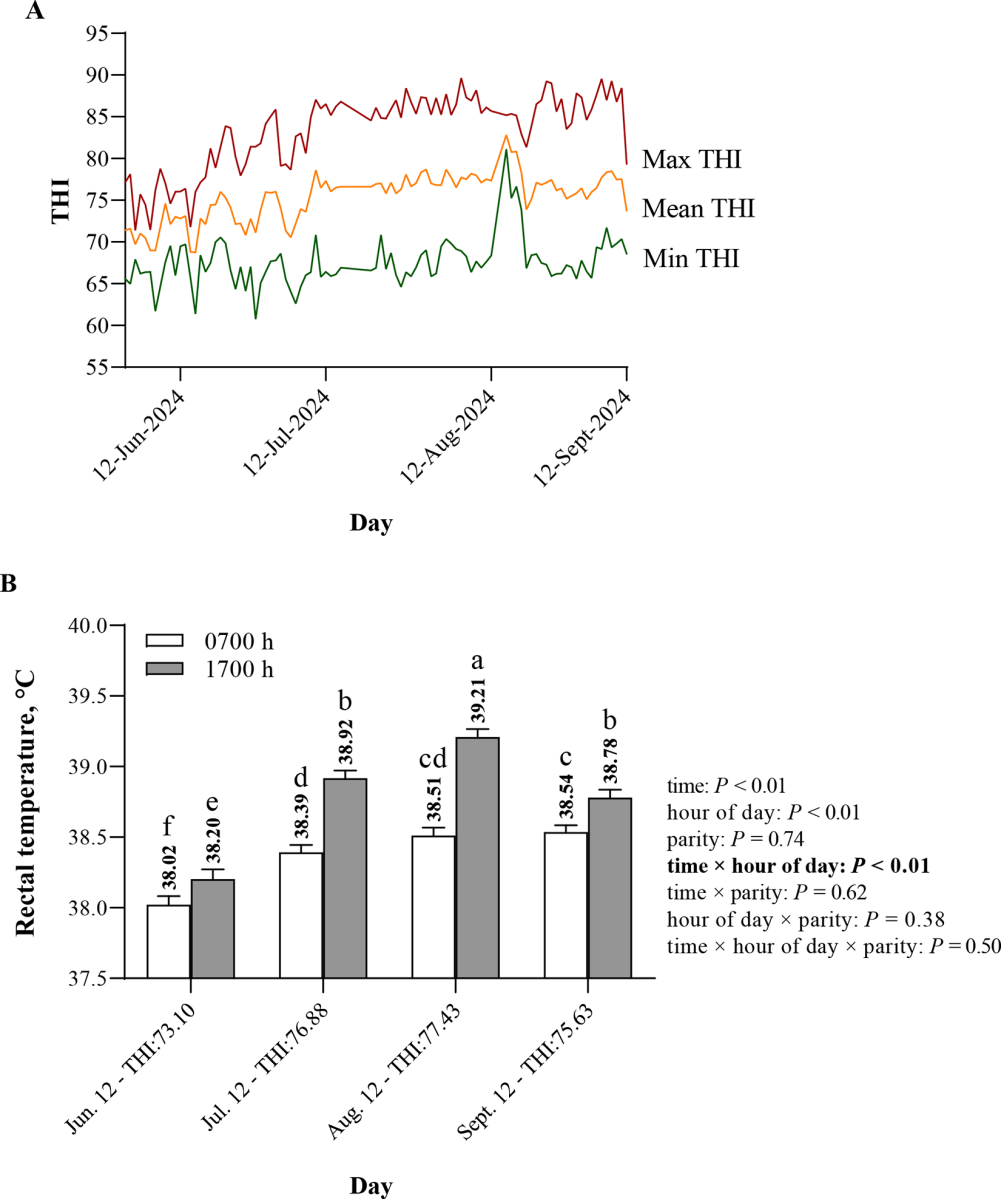
(A) Trend of THI and (B) LSM ± SE of morning (0700 h) and evening (1700 h) rectal temperature (°C) of lactating Cinisara cows over a 90-d period during natural HS exposure in the summer. Boldface indicates this interaction chosen for graphical representation. Bars represent the SEM, and different lowercase letters indicate significant differences (*P* ≤ 0.05). Max = maximum; Min = minimum.

Data were analyzed using SAS software (version 9.4, SAS Institute). Normality of data was checked by using the univariate procedure of SAS. Milk data were analyzed as repeated measurements with the GLIMMIX procedure of SAS. The statistical models included the fixed effects of time (Jun., Jul., Aug., and Sept.), parity (primiparous and multiparous), and their interaction, whereas individual cows were included as random. The model equation was as follows: *Y_ijk_* = *μ* + *T_i_* + *P_j_* + *TP_ij_* + *c_k_* + *ε_ijk_*, where *Y_ijk_* = dependent continuous variable, *μ* = overall mean, *T_i_* = fixed effect of time *i*, *P_j_* = fixed effect of parity *j*, *TP_ij_* = interaction between time and parity, *c_k_* = random effect of *k*th animal (cow), and *ε_ijk_* = residual error. Rectal temperature data were analyzed as repeated measurements with the GLIMMIX procedure of SAS. The statistical models included the fixed effects of time (Jun., Jul., Aug., and Sept), hour of day (0700 h and 1700 h), parity (primiparous and multiparous), and their interaction, whereas individual cows were included as random. The model equation was as follows: *Y_ijkl_* = *μ* + *T_i_* + *H_j_* + *P_k_* + *TH_ij_* + *TP_ik_* + *HP_jk_* + *THP_ijk_* + *c_l_* + *ε_ijkl_*, where *Y_ijkl_* = dependent continuous variable, *μ* = overall mean, *T_i_* = fixed effect of time *i*, *H_j_* = fixed effect of hour (*j*) of day, *P_k_* = fixed effect of parity *k*, *TH_ij_* = interaction between time and hour of day, *TP_jk_* = interaction between time and parity, *HP_jk_* = interaction between hour of day and parity, *THP_ijk_* = interaction between time, hour of day, and parity, *c_l_* = random effect of *l*th animal (cow), and *ε_ijkl_* = residual error. Comparisons with *P* ≤ 0.05 were considered significant. Finally, regressions were performed between the LSM of RT data retrieved from the mixed models and THI values. In detail, segmented regression analysis was conducted using the NLIN procedure to identify the THI breakpoint at which RT began to increase significantly (*P* < 0.05) and abruptly, considering all cows.

To assess heat tolerance in dairy cows, the monitoring of RT and respiration rate are commonly used (Bernabucci et al., 2010) as first indicators for HS detection. Dairy cows maintain the body temperature balancing the heat gain and the heat loss (Hahn, 1999). When skin surface temperature exceed 35°C, cows begin accumulating heat and RT increases (Bakony et al., 2023), causing changes to the physiology of the animal. For this reason, during HS when THI is above 70, the RT increases beyond 39°C (Yan et al., 2021).

In this study, although parity and its interaction with time or hour of the day did not affect RT (*P* > 0.10), a significant increase of RT was observed during the afternoon hours compared with the morning hours (*P* < 0.05), as shown in Figure 1B. During the morning hours, the highest overall RT was registered in August (38.51°C ± 0.06°C; THI = 77.43) and September (38.54°C ± 0.05°C; THI = 75.63), indicating a direct association between RT and THI, whereas the lowest RT was registered in June (38.02°C ± 0.06°C; THI = 73.10). Moreover, the highest RT registered during the afternoon hours was in August (39.21°C ± 0.06°C), where the THI reached 77.48, whereas the lowest RT was registered in June (38.20°C ± 0.07°C) with a THI of 73.10 (Figure 1B). These results showed that RT was consistently higher in the afternoon hours (1700 h) compared with the morning hours (0700 h). This diurnal variation in dairy cows was previously reported and reflects the circadian rhythm of the animal, with a peak in the afternoon and a minimum in the morning, also as a consequence of environmental heat load throughout the day (Theurer et al., 2014).

Moreover, this diurnal fluctuation was relatively small (0.7°C daily fluctuation in August), compared with that reported by Islam et al. (2023) for both heat-susceptible and heat-tolerant feedlot beef heifers in Australia, indicating that heat-susceptible heifers had a 1.5°C daily RT fluctuation (38.24°C to 39.76°C) and heat-tolerant cattle had a 0.87°C daily RT fluctuation (38.13°C to 39.00°C).

The THI threshold was determined when abrupt and significant changes in RT were detected at certain THI value (Figure 2A). A strong correlation (adjusted R^2^ = 0.89) was observed between THI and RT, and a THI threshold was identified for RT. Cinisara cows exposed to natural HS exhibited a THI breakpoint of 77.65 for RT, whereby RT began rising at a rate of 0.04°C for every unit increase in THI below the threshold (Figure 2A), whereas RT began rising at a rate of 0.12°C for every unit increase in THI above the threshold (Figure 2A). Several studies show that Holstein and Brown Swiss dairy cows exhibit a THI threshold between 70 and 75 for an RT of 38.5°C, depending on physiological stage and parity. (Maggiolino et al., 2020; Pinto et al., 2020; Yan et al., 2021). In contrast, Cinisara cows demonstrate a higher THI threshold and a slower increase in RT as THI rises. Previous studies on indigenous local breeds, such as *Bos indicus*, suggested that skin structure could explain the better thermoregulation properties, thanks to sweat gland structure (Mateescu et al., 2023) and skin thickness, facilitating increased blood flow to the skin during HS (Carvalho et al., 1995). Thus, it can be assumed that Cinisara cows are better able to regulate body temperature in response to HS compared with Holstein and Brown cows, probably because they are able to maintain the balance between the ability to dissipate heat through skin and lower their metabolic rate. This is also demonstrated by the maintaining of RT in the physiological range in both morning and evening hours, without hyperthermia occurring even during the summer months. However, further studies on the structural characteristics of the skin and hair of Cinisara cows could help to clarify their enhanced heat dissipation mechanisms.

**Figure 2:**
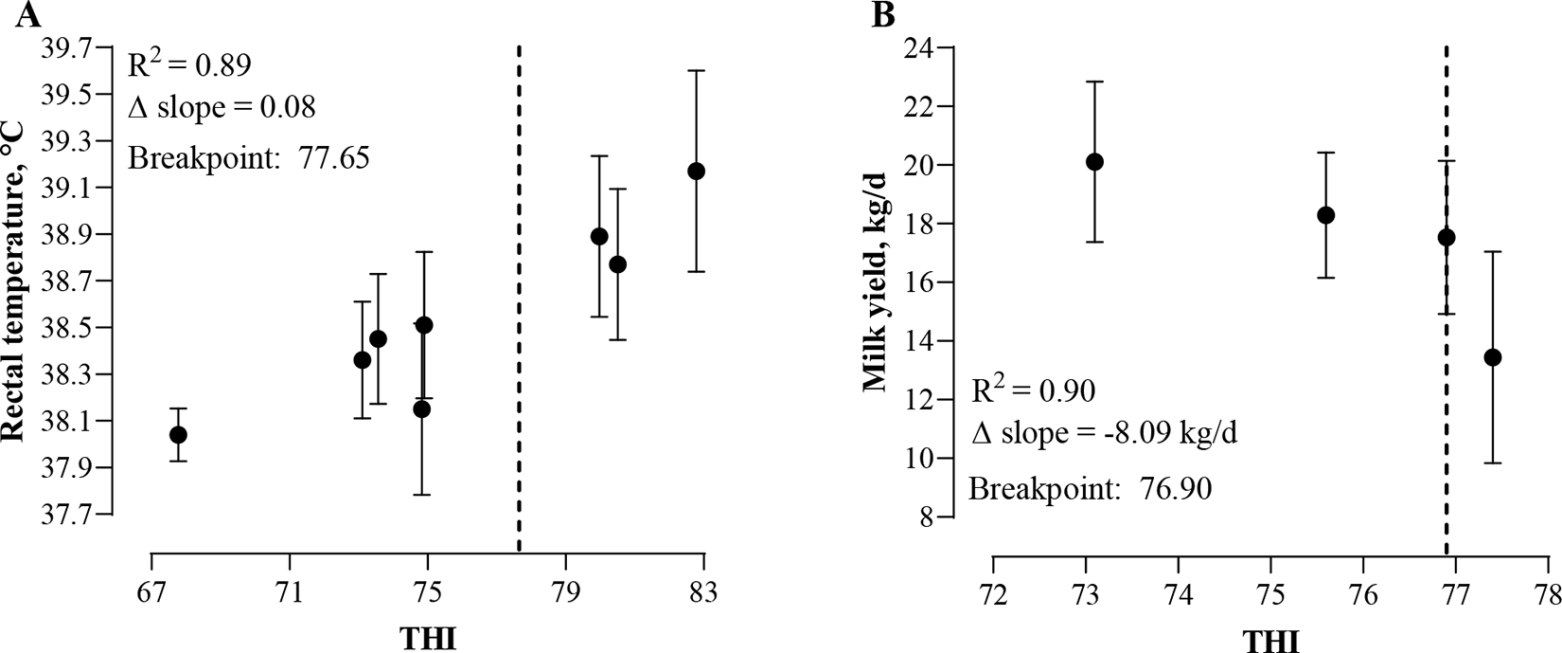
Nonlinear segmented regressions of (A) rectal temperature (°C, LSM ± SD) and (B) MY (kg/d, LSM ± SD) in lactating Cinisara cows relative to THI over a 90-d period during natural HS exposure in the summer. Vertical dashed lines indicate the breakpoint at which rectal temperature and daily MY changed significantly and abruptly. Δ Slope represents the change in slope between the slope of the data before breakpoint (b_1_) and the slope of the data after breakpoint (b_2_).

The THI threshold was determined also for MY (Figure 2B). Despite the limited number of time points (n  =  4), segmented linear regression applied to average daily milk production data identified a breakpoint at THI = 76.9 (Figure 2B). Before the threshold, the decline in MY was moderate (−0.69 kg/d per THI unit; 95% CI: −1.14 to −0.23 kg/d), whereas beyond this point, milk production dropped considerably (−8.77 kg/d per THI unit; 95% CI: −11.84 to −5.71 kg/d). The difference in slope (Δ = −8.09 kg/d; 95% CI: −11.58 to −4.59 kg/d) reflects a marked reduction in milk output at high THI levels (Figure 2B). The model showed a strong correlation (adjusted R^2^ = 0.90). Regarding milk production and its composition response, parity and its interaction with time did not affect milk data (*P* > 0.10). Overall, MY was significantly affected by time (*P* < 0.01), reflecting the trend of THI during the 90-d of the monitoring period (Figure 3A). Specifically, MY was on average 20.11 ± 0.70 kg/d in June (THI = 73.10), decreased to 17.53 ± 0.57 kg/d in July (THI = 76.88), reached its lowest value of 13.43 ± 0.5 kg/d with the highest THI in August (THI = 77.43), and then increased in September to 18.29 ± 0.62 kg/d when THI reached lower levels (THI = 75.63). Previous studies on Holstein cows defined the threshold for milk production at ∼68, where cows started to significantly reduce the MY, with further declines at higher THI values (74 or 76; Bernabucci et al., 2014; Heinicke et al., 2018). However, several studies have evaluated a different MY responses among breeds during HS. For example, Collier et al. (1981) suggested that Jersey cows are more heat tolerant than Holstein cows in relation to milk production, and Smith et al. (2013) observed an increase in milk production in Jersey and a decrease in Holstein cows under HS. Our recent study revealed that mid-lactating Fleckvieh cows showed a THI threshold for milk yield of ∼73, where cows started to significantly reduce the MY at a rate of ∼1.5 kg/d at THI above 73 (Amato et al., 2025). This result suggests that this local breed may have developed mechanisms to better tolerate high temperatures, maintaining milk production until higher THI (∼77), and thus having better resistance to HS. Although a reduction of ∼8 kg/d in MY beyond the THI breakpoint may appear excessive, it is important to note that this drop occurs only after exposure to a particularly high THI. In comparison, most conventional dairy breeds begin to show declines in milk production at THI values between 69 and 73. The Cinisara breed, by contrast, is able to maintain stable MY up to a THI of 77 (even though Cinisara is a low-producing breed). Indeed, below the threshold, MY decreased only marginally, with a slope of −0.69 kg/d per unit of THI (95% CI: −1.14 to −0.23 kg/d), suggesting a higher tolerance to HS. Thus, although the overall reduction is similar compared with other milk-specialized breeds, the Cinisara appears to delay the negative effects of heat, highlighting its adaptive advantage under hot environmental conditions. However, it is noteworthy to consider that overall, the MY of Cinisara cows is lower compared with Holstein cows (on average 3,700 kg of milk per lactation vs. 10,000 kg, respectively); thus, the lower MY and therefore the lower metabolic heat contribute to reduced susceptibility to HS. Environmentally induced hyperthermia not only affects overall milk production, but also milk composition, decreasing fat, protein, and lactose content (Mylostyvyi and Chernenko, 2019; Florio et al., 2022).

**Figure 3:**
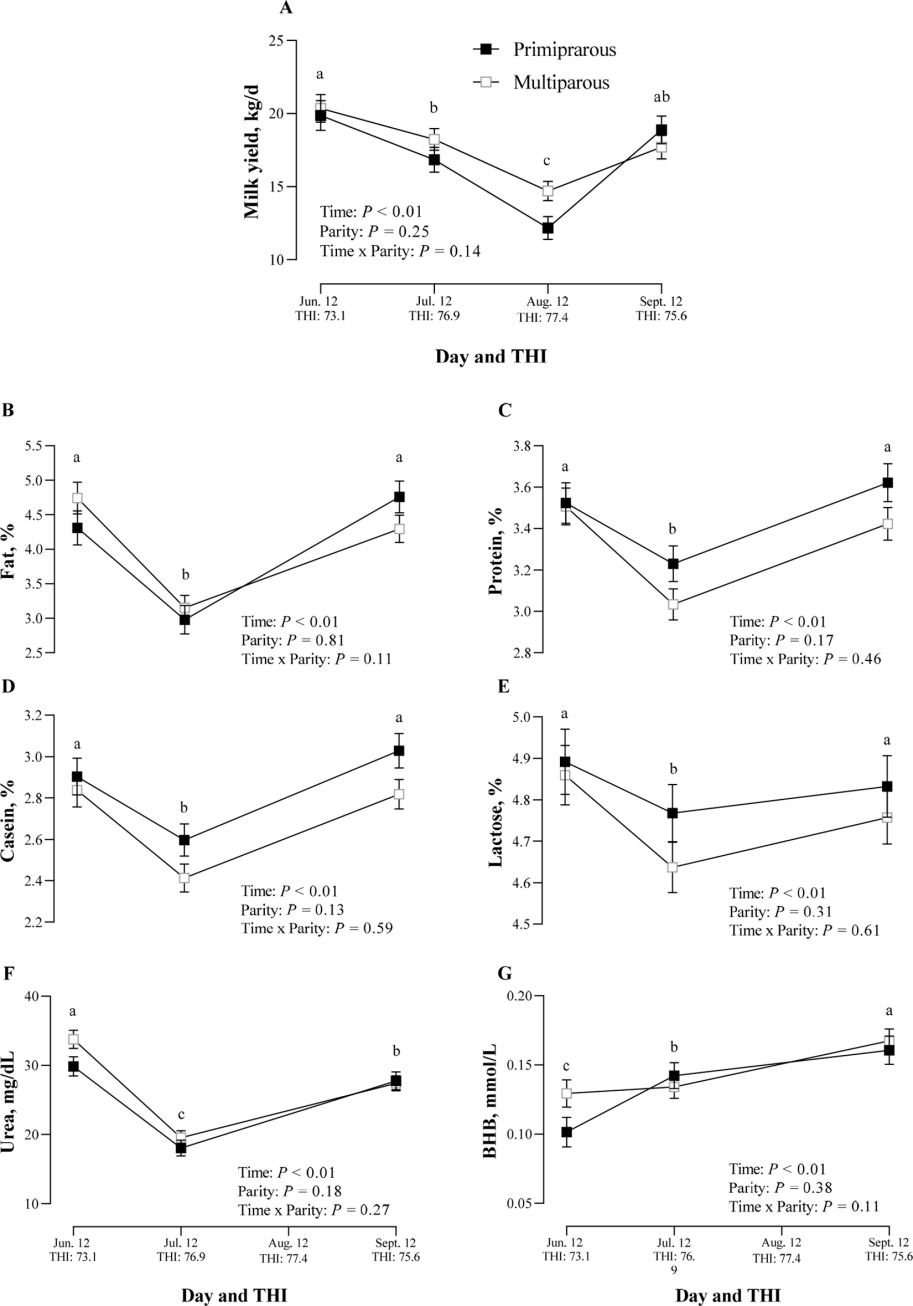
(A) Milk yield and (B–G) milk composition (LSM ± SE) in lactating Cinisara cows over a 90-d period during natural HS exposure in the summer. Bars represent SEM, and different lowercase letters indicate significant differences among time points (*P* ≤ 0.05).

Consistently with this, results on Cinisara milk quality (Figures 3B–G), exhibited significant variations (time; *P* < 0.01) over time and THI, with a decrease in milk components during the hottest period of the trial in July, when THI was above 76. In fact, in July (THI = 76.88) a decrease in milk fat (Jun.: 4.53% ± 0.17%; Jul.: 3.06% ± 0.14%; Sep.: 4.52% ± 0.15%), protein (Jun.: 3.51% ± 0.07%; Jul.: 3.13% ± 0.06%; Sep.: 3.52% ± 0.06%), casein (Jun.: 2.87% ± 0.06%; Jul.: 2.50% ± 0.05%; Sep.: 2.92% ± 0.06%), lactose (Jun.: 4.88% ± 0.05%; Jul.: 4.70% ± 0.05%; Sep.: 4.79% ± 0.05%), and urea (Jun.: 31.81% ± 0.96%; Jul.: 18.80% ± 0.76%; Sep.: 27.59% ± 0.85%) content was observed compared with June (THI = 73.10) and September (THI = 75.63), where their levels were higher. The decrease in milk fat is mainly related to the decrease in DMI (Collier et al., 2006), which leads to a decrease in the ruminal production of acetate, a precursor for milk fat (Kim et al., 2022). However, it is noteworthy that after the peak of THI, the milk fat content of Cinisara cows reached greater levels. Similarly, the decrease in milk protein and urea during the peak of THI could be related to the decrease in DMI, but this mechanism can also be compromised by the alteration in amino acid circulation (Gao et al., 2017), as amino acids are redirected to support maintenance of metabolism rather than milk synthesis (Cartwright et al., 2023). Indeed, Cowley et al. (2015) pointed out that these reductions in milk protein of heat-stressed cows appear to be a result of specific downregulation of mammary protein synthetic activity, exacerbated by the increased protein turnover and AA competition between casein and structural proteins. In addition to mammary gland intrinsic mechanisms, heat stress–induced milk protein reductions might be the result of limitations in the precursor supply caused by the reduction in mammary blood flow. When dairy cows rely predominantly on pasture, long periods of dry weather during summer and HS can reduce both overall biomass intake and nitrogen intake from forage. This alteration substantially affects ruminal protein metabolism, resulting in a decreased supply of precursors for milk protein synthesis due to a reduction in rumen microbial protein production (Bernabucci et al., 2015). This decline in microbial protein synthesis may partly explain the observed reduction in milk urea levels during the hottest periods. In contrast, previous studies involving cows fed TMR have reported increased blood and milk urea levels under induced and naturally occurring HS conditions (Bernabucci et al., 2014; Gao et al., 2017; Hou et al., 2021; Maggiolino et al., 2025), likely originating from both inefficient rumen ammonia incorporation into microbial protein and hepatic deamination of AA mobilized from skeletal muscle (Bernabucci et al., 2015). Thus, we speculate that the urea metabolism of Cinisara cows raised in an extensive system, where pasture is the primary nutritional source, is more susceptible to nutritional influences than to the direct effects of HS.

A similar pattern of metabolic adaptation was observed in energy metabolism, as evidenced by changes in BHB levels. Indeed, BHB content was conversely lower in June (Jun.: 0.12 ± 0.01 mmol/L), and then significantly increased during the peak of THI in July (Jul.: 0.14 ± 0.01 mmol/L) until reaching its highest levels in September (Sep.: 0.16 ± 0.01 mmol/L). A previous study also observed a rise in BHB in cows experiencing HS, reflecting fat mobilization for reducing energy intake (Ishida et al., 2024). This study observed that during HS, milk BHB increased by 0.1 to 0.3 mmol/L, compared with cooled cows, reflecting our result. However, the significant increase of BHB in September can be related to the increase in MY, which raises the energy requirements of the cows. However, the role of pasture cannot be excluded. Indeed, given that Cinisara cows depend primarily on natural pasture for their nutritional intake, the observed decline in MY and its composition during the hotter periods (July and August) could be attributed not only to the direct effects of HS, but also to a substantial reduction in both the biomass and the energy content of the available forage. As temperatures rise and drought conditions intensify, the quality of the pasture tends to deteriorate, with lower levels of digestible nutrients and energy, thereby compromising the animals' ability to meet their physiological requirements for sustained milk production. These results suggest that Cinisara cows exhibit good tolerance to HS, maintaining RT within physiological range even during the peak of summer. Although milk production and quality were negatively affected by HS, they started to being triggered at higher THI levels. Moreover, the impacts were less pronounced compared with those observed in Holstein and Brown cows in previous studies. Thus, the better resilience and adaptability to harsh environmental condition of Cinisara cows can be a resource for the future of dairy farming in the Mediterranean area. Further studies are needed to explore the genetic and physiological mechanisms involved in the heat tolerance of Cinisara cattle in order to preserve and promote biodiversity conservation.
